# Translation, cultural adaptation and validity assessment of the Dutch version of the eHealth Literacy Questionnaire: a mixed-method approach

**DOI:** 10.1186/s12889-023-15869-4

**Published:** 2023-05-30

**Authors:** Charlotte C. Poot, Eline Meijer, Marjolein Fokkema, Niels H. Chavannes, Richard H. Osborne, Lars Kayser

**Affiliations:** 1 Department of Public Health and Primary Care, Leiden University Medical Centre, Leiden, The Netherlands; 2 National eHealth Living Lab (NeLL), Leiden University Medical Centre, The Netherlands, Leiden, The Netherlands; 3 Methodology and Statistics Research Unit, Institute of Psychology, Leiden University, Leiden, The Netherlands; 4 Centre for Global Health and Equity, Swinburne University of Technology, Melbourne, VIC Australia; 5 Department of Public Health, University of Copenhagen, Copenhagen, Denmark

**Keywords:** eHealth, Health literacy, Digital health, Questionnaire design, Translation, Psychometrics

## Abstract

**Background:**

The digitalization of healthcare requires users to have sufficient competence in using digital health technologies. In the Netherlands, as well as in other countries, there is a need for a comprehensive, person-centered assessment of eHealth literacy to understand and address eHealth literacy related needs, to improve equitable uptake and use of digital health technologies.

**Objective:**

We aimed to translate and culturally adapt the original eHealth Literacy Questionnaire (eHLQ) to Dutch and to collect initial validity evidence.

**Methods:**

The eHLQ was translated using a systematic approach with forward translation, an item intent matrix, back translation, and consensus meetings with the developer. A validity-driven and multi-study approach was used to collect validity evidence on 1) test content, 2) response processes and 3) internal structure. Cognitive interviews (*n* = 14) were held to assess test content and response processes (Study 1). A pre-final eHLQ version was completed by 1650 people participating in an eHealth study (Study 2). A seven-factor Confirmatory Factor Analysis (CFA) model was fitted to the data to assess the internal structure of the eHLQ. Invariance testing was performed across gender, age, education and current diagnosis.

**Results:**

Cognitive interviews showed some problems in wording, phrasing and resonance with individual’s world views. CFA demonstrated an equivalent internal structure to the hypothesized (original) eHLQ with acceptable fit indices. All items loaded substantially on their corresponding latent factors (range 0.51–0.81). The model was partially metric invariant across all subgroups. Comparison of scores between groups showed that people who were younger, higher educated and who had a current diagnosis generally scored higher across domains, however effect sizes were small. Data from both studies were triangulated, resulting in minor refinements to eight items and recommendations on use, score interpretation and reporting.

**Conclusion:**

The Dutch version of the eHLQ showed strong properties for assessing eHealth literacy in the Dutch context. While ongoing collection of validity evidence is recommended, the evidence presented indicate that the eHLQ can be used by researchers, eHealth developers and policy makers to identify eHealth literacy needs and inform the development of eHealth interventions to ensure that people with limited digital access and skills are not left behind.

**Supplementary Information:**

The online version contains supplementary material available at 10.1186/s12889-023-15869-4.

## Background

### Digitalization of Healthcare

The use of digital technologies for health, also called eHealth, is revolutionizing the way we diagnose, treat and manage health and disease. eHealth, defined as “the use of information and communications technology in support of health and health-related fields” spans a range of different digital health technologies and services, including smartphone apps, remote monitoring, smart wearables, patient portals and electronic patient records [1]. Given the wide application and spectrum of eHealth, eHealth is often presented as a solution to relevant healthcare challenges, including challenges posed by the ageing population, the increased number of chronic and multi-morbidities and the growing resource gap [2, 3]. As a result, eHealth has been stimulated and has shaped the way people engage with their health and how information is exchanged and shared between patients, healthcare providers and across health ecosystems.

### eHealth literacy

This changing healthcare landscape has added complexity in the way community members, healthcare professionals and digital technologies interact. For example, healthcare portals and telehealth systems allow people to remotely communicate with healthcare professionals and caregivers, electronic health records based on cloud storage allow patients to manage diagnostic data with clinicians, and wearables and apps can help people to self-manage their condition. However, this increased complexity requires additional skills and competences from people using eHealth, including patients and people without a medical diagnosis. In the early days of the internet (web 1.0) the additional set of needed skills to navigate the web was introduced as eHealth Literacy: “the ability to seek, find, understand, and appraise health information from electronic sources and apply the knowledge gained to addressing or solving a health problem.” However, with the increased complexity of the digital health landscape scholars have called for a more comprehensive view and included elements related to users’ cognitive skills, communication elements, social and cultural context or system level attributes [4–6]. Since the web 1.0, eHealth literacy and its association with health outcomes has been investigated extensively [7, 8]. However, eHealth literacy as evolved concept in the new digital (health)landscape and its impact on health, is a relatively new area that needs to be further investigated [9, 10].

### eHealth, covid-19 pandemic and digital divide

eHealth literacy has gained attention with the accelerated uptake of eHealth due to the covid pandemic, mainly with the use of telehealth and remote monitoring systems [11–13]. While large scale studies are lagging behind, smaller scale studies indicate that people who have low digital literacy and health literacy have difficulties comprehending and navigating through the information on the internet, downloading and using teleconsultation software, and understanding the already complex security safeguards and privacy policies necessary to effectively interact with telehealth devices [14, 15]. Also, studies evaluating use of telehealth during the pandemic observed a lower usage among people who were lower educated [16, 17]. This so-called digital divide in which digital systems are more frequently used by people with higher education is of particular concern as people with lower education and fewer resources generally more often need ongoing medical care [11, 18]. As such, academics have expressed concerns that ongoing digitalization of the health landscape may ultimately result in increasing health inequities and exclusion of those who are digitally disadvantaged [15, 19, 20]. This issue is not new [20–22]. In fact, the WHO has recognized the digital divide with risk of digital exclusion and unequal access as one of the biggest challenges posed by the digital transformation of healthcare [23].

### Measuring eHealth literacy

Adequate assessment of eHealth literacy is instrumental in bridging the digital divide. Over the years, several instruments have been developed to measure eHealth literacy [24–28], with the eHealth Literacy Scale (eHEALS) the most commonly used [25, 28] due to its early development. The eHEALS measures perceived skills in finding, evaluating, and applying electronic health information related to health problems using first-generation internet based health services [25, 29]. The instrument does, however, not fit with the evolving concept of eHealth literacy and today’s broad scope of digital technologies which requires a wider range of competences [30, 31], like entering data in patient portals or health apps on a smartphone [30, 32]. The Digital Literacy Instrument (DLI) was developed to overcome these limitations. Data collected have shown validity and reliability in a Dutch sample [24, 28]. However, the instrument is performance-based and covers individual skills in digital health technology use, without capturing broader interactions with health technologies and services, including motivation to engage with digital health technologies.

### The eHealth Literacy Questionnaire

To overcome the shortcomings of the eHEALS, the eHealth Literacy Questionnaire (eHLQ) was developed. The 35-item eHLQ is based on the eHealth Literacy Framework (eHLF), developed in 2012 with patients and medical professionals during a systematic concept mapping process [33]. This framework includes individual factors that are necessary to use eHealth (e.g., engagement in own health), system factors (e.g., access to digital services that work) and user–system interaction factors (e.g., motivation to engage with digital services). The constructs were conceptualized into seven conceptually distinct dimensions that present a multifaceted understanding of eHealth literacy and are measured by the eHLQ [34, 35]:

1. Using technology to process health information (five items)
2. Understanding of health concepts and language (five items)
3. Ability to actively engage with digital services (five items)
4. Feel safe and in control (five items)
5. Motivated to engage with digital services (five items)
6. Access to digital services that work (six items)
7. Digital services that suit individual needs (four items)

Each item is scored on a 4-point scale (strongly disagree, disagree, agree, strongly agree). The questionnaire was developed in Danish and English simultaneously “to support researchers, developers, designers, and governments to develop, implement, and evaluate effective digital health interventions” [35]. As such, the eHLQ has been used to understand people’s interaction with eHealth devices [34, 36, 37], to evaluate the association between eHealth literacy and health outcomes [38] and to inform the adaptation of health technologies [39]. The eHLQ has been shown to have strong construct validity, reliability, is easy to use [35, 40, 41] and is intended to be used by policy makers, eHealth developers and researchers. It can be used in a wide range of settings including community health or hospitals and was designed for self-administration by pen and paper or by interview to ensure inclusion of persons with visual, reading or other difficulties. The questionnaire is supported by an instruction page including an explanation of terms used in the questionnaire.

### Validity assessment

Use of a questionnaire in a novel linguistic setting requires translation, cultural adaption and validity assessment of the questionnaire [42], in order to determine that it’s properties have not been compromised and are equivalent to the original instrument. In the field of questionnaire validity testing, there is a growing acceptance of the view that the validity testing of self-reported instruments is as an accumulation and evaluation of different sources of validity evidence [43]. As such, validation includes several supportive arguments on validity, rather than relying on factor analysis or regression analysis only [44, 45]. The standards for Educational and Psychological Testing (in short, ‘the Standards’) are a set of guidelines which can be used to guide evaluation of validity evidence [46]. The Standards, considered best practice in the field of psychometrics, proposes five sources of evidence: 1) test content; 2) response process (i.e. respondents’ cognitive processes when responding to the items, such as understanding the instructions, interpreting the items as intended); 3) internal structure (i.e. the extent to which the items conform to constructs and constructs are conceptually comparable across subgroups and with repeated administration); 4) relations to other variables, and 5) consequences of testing (i.e., the robustness of the proposed instrument use including intended benefits, indirect effect and unintended consequences). By using evidence on content, response and internal structure as a framework, we build upon previous validation studies of the original instrument and systematically use different sources of validity. We used this evidence to inform the development of a Dutch version of the eHLQ and assess its properties. Relations to other variables [29] and consequences of testing [4] remain beyond the scope of this study.

### Relevance and study aim

In line with global developments, the Netherlands is transforming its healthcare system and investing in various forms of eHealth. Accelerated by the covid pandemic, eHealth is increasingly adopted and implemented across various disciplines in primary care [47] and secondary care [48, 49]. Despite eHealth gaining ground, a comprehensive Dutch person-centered instrument to measure eHealth literacy is lacking. Hence, the aim of this study was to translate and culturally adapt the original eHLQ into a Dutch version, and to examine validity of the translated instrument.

## Method

### Overall study design

**Fig. 1:**
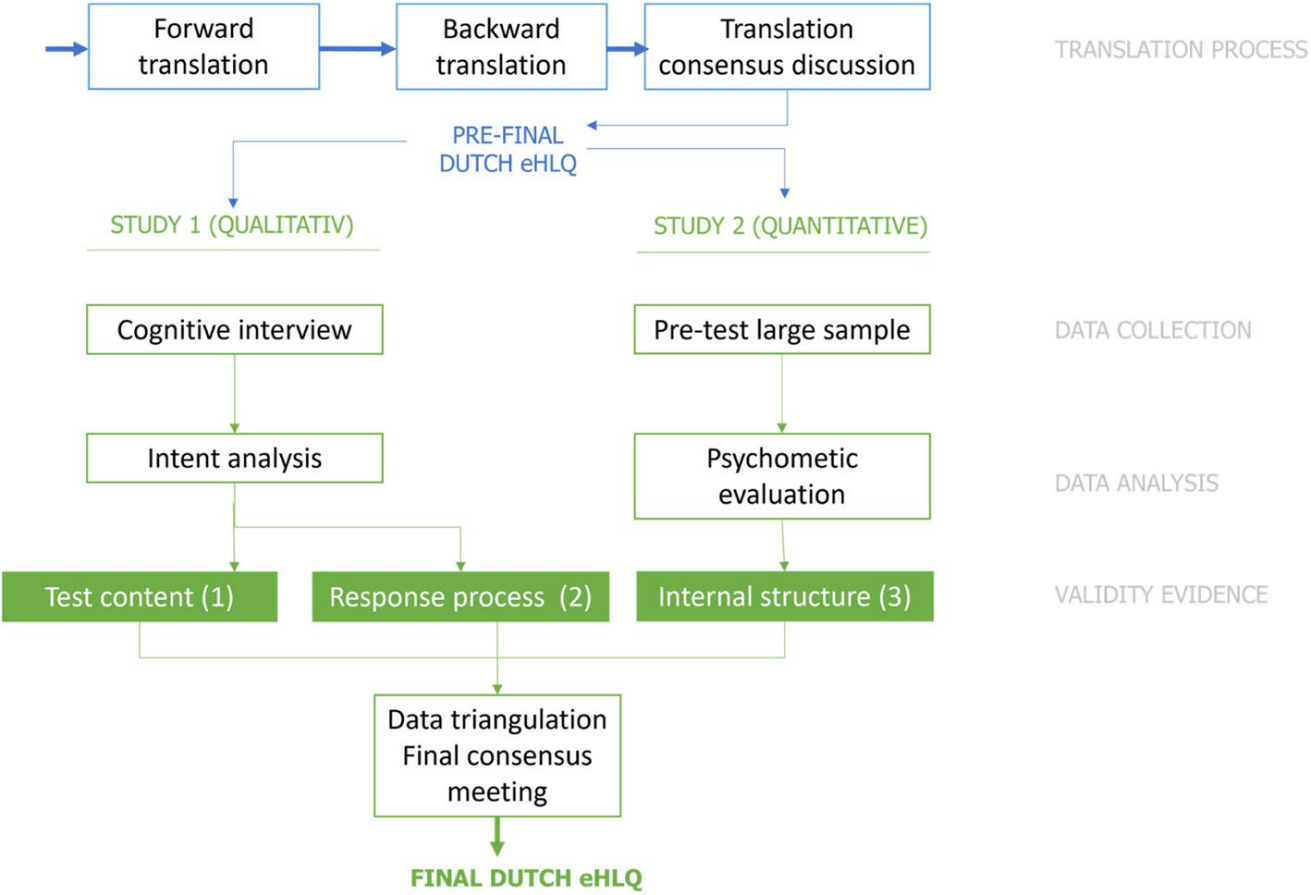
Schematic overview study design: a concurrent mixed-method design

In this paper we report on the translation of the original eHLQ, and two studies performed to assess the initial validity evidence that was used to inform the final translation and cultural adaption. Our research was guided by the Standards to assess validity evidence. Figure 1 provides a schematic outline of the study design and the relation between the two studies. In Study 1, evidence on 1) content validity and 2) response process was collected using cognitive interviewing. In Study 2, the initial eHLQ was tested in a large sample and evidence on 3) internal structure validity was collected. Studies in cross-cultural adaptation of instruments often first perform cognitive interviews, then change wordings or phrasings, and subsequently evaluate psychometric properties of the final instrument [50, 51]. We instead performed Study 1 and 2 simultaneously, which allowed us to use results from both studies in the decision on item revision and final translation, instead of changing items based on cognitive interview data only. In the final consensus stage, more weight was given to the cognitive interview data over psychometric data, considering the richness of qualitative data. As such, this study had a nested mixed-method design [52, 53]. We formulated validity evidence arguments per source of validity evidence. The Dutch and other translations of the eHLQ are available upon request from the original authors (LK, RHO) [54].

### Translation process

The original English eHLQ was translated into Dutch following the Translation Integrity Procedure (TIP) set up by the developers. The TIP is a documented systematic translation method that includes the careful specification of descriptions of item intent [55]. It includes an item intent matrix describing the intended meaning and conceptual basis of each individual item, and a translation management grid that can be used by the translation team to guide the translation process. Both documents were used to track ambiguities, guide discussions on the nuances of item meanings and identify focus points for further evaluation. The steps are detailed below.

#### Forward translation

Two bilingual translators independently translated the original English eHLQ to Dutch, following the item intent guide. The first translator (CP), affiliated to the Leiden University Medical Center and the National eHealth Living Lab (NeLL) was knowledgeable about health and eHealth. The second translator (AR), a certified translator with rich expertise in medical research translation. The individual versions were compared and consensus on an initial translation was reached through discussion.

#### Back translation

The initial translation was then translated back to English by an independent translator (MS) who was blinded to the original English version of the questionnaire. The back-translator was a native English speaker, fluent in Dutch and a linguistic expert.

#### Translation consensus discussion

During a first translation consensus team meeting, the back and initial forward translations were compared against the original questionnaire and the item intent. The consensus team was composed of both forward translators (CP and AR), the backward translator (MS), the developer (LK) and an expert team, including two bilingual representatives (EM and PH), both working in health innovation, and a field worker (IM) experienced in questionnaire administration. Any ambiguities and discrepancies were documented and resolved during the meeting. The consensus meeting resulted in a version that was ready for pre-testing and generated a list of items to examine more closely during pre-testing.

## Study 1: qualitative study

The first study aimed to assess validity evidence on test content and response process. Cognitive interviews were performed with a diverse sample of individuals who were considered potential future respondents of the Dutch eHLQ.

### Method study 1

#### Participants

Fourteen people participated (see Additional file 1 for demographics). This sample size was deemed sufficient to identify the most important problems [56]. Inclusion criteria were able to read and express their thoughts in Dutch, and being 18 years of age or older. The Dutch eHLQ is meant to be used among the general Dutch population. While the ‘general Dutch population’ is an ambiguous definition we used purposive sampling, to ensure a wide variation in terms of demographics, health condition and prior experience with eHealth. Participants were recruited via various channels including posters in public areas of Leiden University Medical Center, various sports clubs in the region, patient organizations and the a co-author’s personal network.

People interested in participation were contacted by telephone to confirm their interest, to explain the study, and to schedule the interview. The interview was held at a quiet location (mostly the participant’s home). Prior to data collection, written informed consent was collected.

#### Data collection

##### Cognitive interview

Cognitive interviews were held to assess the 1) test content and 2) response process. We adopted the validity arguments formulated by Cheng et al. in a validity study on the original eHLQ [41]. The validity arguments for test content included themes, wording, format of items, administration and scoring. Assessment of the response process includes assessing whether the items were understood by the respondents as intended by the developers, whether items were understood similarly across subgroups, and whether the number of items, response format and instructions were appropriate.

The cognitive interviews were performed by an experienced qualitative researcher (CP) and a researcher trained in cognitive interviewing, and lasted between 1.5–2 h. The interviews followed a think-aloud approach in which respondents were asked to verbalize their thoughts while completing the questionnaire. This helps to understand the mental processes of respondents as they interpret questions and formulate answers, with minimal interference of the interviewer [57]. In addition, problems regarding memory retrieval, ambiguities or unclear perspectives can be elucidated. The think-aloud exercise was complemented by spontaneous and scripted probing [58]. Spontaneous probes were used based on a respondent’s response such as signs of hesitation (e.g., responses to certain items taking longer than to other items) and included questions such as ‘I saw you hesitate while answering item [X]. Could you explain why?’. Scripted probing helped to explore items which needed further exploration according to the consensus teams. Scripted probes were ‘what does [word or phrasing] mean to you?’. The subsequent structured part of the interview was guided by a manual containing items and scripted probes. The combination of a respondent-driven approach (think aloud) and an interviewer-driven approach (scripted probing) shows the cognitive processes of the interviewee, while also being able to reflect on ambiguous or problematic items in detail [58, 59]. To minimize the cognitive burden on participants, the interview was divided in 3 parts. Participants first completed the first 11 items, thinking aloud, and then responded to scripted probes. This process was repeated for the next two sets of 11 items. Participants received a 20-euro gift card for their participation.

Debriefing sessions were held among the researchers to reflect on the interviews and the interview guide, and to include emergent probes (scripted probes) based on previous interviews. For example, if multiple participants felt an item was ambiguous or unclear, a scripted probe was added to the interview guide to examine the item further.

#### Data analysis

Interviews were analyzed through an item-to-item review, guided by the first three stages of Hacomb’s six-stage model [60]. We audio recorded the interview and took notes during the interview (step 1), held debriefing sessions after a set of three interviews (step 2), and had three researchers familiarize themselves with the data (step 3). Responses relevant to item interpretation were then transcribed, organized per item, compiled for all participants and reviewed item-per-item. For the item-per-item review, responses were compared to the item-intent guide, carefully examining whether the items were understood as intended. Item response problems were coded following the problem item classification coding scheme by Knafl and colleagues, classifying problematic items based on the type of problem encountered [61]. The coding scheme included the following code categories: (a) limited applicability; referring to a comment on groups of people or situations for which the item is or would not be appropriate (b) unclear reference; referring to lack of clarity regarding what aspect, condition or situation the item is intended to address, (c) unclear perspective; pointing towards problems in clarity regarding the perspective from which the items should be answered and (d) problems with wording or tone. We also assessed clarity of response options, recall problems and resonance with local worldview [57].

### Results study 1

#### Cultural adaptation during translation process

During the translation process several items required cultural adaption to appropriately reflect their meaning in Dutch. First, English expressions such as ‘make technology work for me’, ‘works together’ ‘find my way’ and ‘have good conversation’ lack meaningful direct translation into Dutch. Alternative translations were tested and included as scripted probing during the cognitive interviews. Second, nuances between ‘sure’ and ‘confident’ and ‘good conversations about health’ and ‘take part in conversations about health’ were discussed and explored further during the cognitive interviews, to ensure content validity and sufficient contrast between the items. Third, back translation deviated somewhat from the original wording, as more common phrasings were preferred to literal translations (i.e., ‘those who need it’, ‘measurements about my body’, ‘organise’). Content validity was explored using scripted probes. Lastly, cultural adaptation was needed for some terms included in the terminology list.

#### Results on test content and response process

Fourteen cognitive interviews were held. The age of the participants ranged between 27 and 73 years old (median age 61); ten participants were male; six were considered low educated; six indicated that they did not have affinity with digital technology. Their previous eHealth experiences were mainly smartphone and computer use. Some also had a digital blood pressure device or used an online patient portal from their healthcare provider (see Additional file 1 for an overview of respondent’s characteristics).

Respondents’ comprehension of the Dutch eHLQ was satisfactory as they were able to adequately comment on their responses with respect to each item. Respondents generally understood the response options and were able to distinguish among them, although some participants desired additional scoring option ‘not applicable’ for items referring to ‘problems with my health’ and ‘all the health technology I use’.

Respondents commented on limited applicability, unclear reference and problems with wording or tone for 12 items. In addition, a problem in resonance with local worldviews was found in four items. No problems were found regarding unclear perspective, recall problems or clarity of response options (see Additional file 4).

##### Wording or tone

From the items marked for additional exploration based on the cultural adaption in the translation phase, four items were classified as problematic due to problems with wording or tone. The Dutch word for the word ‘organise’ (NL ‘ordenen’) in the item ‘organise my health information’ was confused with ‘sorting things in/on colour or shape’. Other wording problems included ‘take care of my health’, ‘work together’ and ‘monitor’.

##### Limited applicability

Limited applicability was seen in two ways 1) items concerning health problems (i.e., people without health problems), 2) items on use of digital health services (i.e., people not using digital health services).

##### Unclear reference

Four items were marked by an unclear reference. Participants were unsure whether an item referred to their own health or health in general (i.e., ‘health problems in general or my health problems’; item 11 and 20). The majority also struggled with the word ‘*nuttig*’ in item 6 (English translation ‘work for me’), indicating that it was too vague. Despite the terminology list, participants who were less familiar with eHealth were unsure what health technology and health technology services included, and wondered whether it also included telephone and email.

##### Resonance with local worldviews

Cognitive interviews also revealed a problem in resonance with worldviews in 8 items. Participants who frequently used eHealth privately or professionally, expressed their wish to have ‘all technology work together’ (item 23) and have information about their health always ‘available to those who need it’ (item 3), but had had no such experience. Participants less familiar with eHealth had difficulties responding to these items and with understanding the items within their references and knowledge on digital health technologies. Some participants also had difficulties responding to three items from domain 7 on ‘digital services that suit individual needs’ (items 28,31 and 34) as they found it difficult to envision how technology services can adapt to someone’s skills. Only two respondents, who were professionally involved in eHealth, responded with thinking of ‘self-learning machines’ and ‘artificial intelligence’, thereby voicing the items’ intent most closely. The dissonance with local worldviews can point to differences in how items of the eHLQ are interpreted across subgroups. Differences were mainly observed based on having a current diagnosis, previous eHealth experience and educational level.

Besides the above-mentioned issues, we noted that all respondents remarked on similarity of items 19 and 20, and items 22 and 30. Although there were no intent or content problems (i.e., respondents noted the nuance differences), some respondents noted that having very similar items could cause irritation and advised to include a remark on having similar items in the instructions.

## Study 2 – quantitative study

Study 2 was performed to perform psychometric evaluation and assess internal structure of the pre-final eHLQ. The pre-final eHLQ was administered among the 1650 people participating in the FitKnip study. The size of the sample was conform the sample size requirements for factor analysis and deemed sufficient [62].

### Method Study 2

#### Participants

The eHLQ was administered online among participants of the FitKnip study, as part of its baseline measurements. The FitKnip study evaluated the use of a digital health budget as an innovative way to improve population health. Participants received a digital health budget of 100 euro to purchase preselected mobile or web applications offered on the online FitKnip library. People were recruited via municipality teams and various institutions, including healthcare insurance companies, an organization for vital and healthy neighbourhoods, and patient organizations. People had to be 18 years or older, able to understand, read, and speak the Dutch language and have access to the internet, but no other in- or exclusion criteria were applied.

#### Data collection

The eHLQ was included in a battery of six questionnaires on mental and physical health, general wellbeing and health awareness, and administered online among the FitKnip participants. Participants provided digital informed consent for the entire study prior to completing the questionnaire. The questionnaire battery was sent to 2562 participants and returned by 1650 respondents within 1 month (response rate 64%). There was no missing data among the 1650 returned eHLQ questionnaires. Participants received access to their digital health budget after completing all six questionnaires. Within the study demographic data, age, gender and educational background were collected. Educational level was categorised as low (no education to lowest high school degree), middle (vocational training to highest high school degrees) and high (university of applied sciences degree and research university degree). People were also asked to indicate whether they had a current medical, physical or psychological diagnosis.

#### Data analysis

##### Preparatory analyses

Descriptive statistics were used to describe the means and standard deviations of individual items, and to identify floor or ceiling effects. Internal consistency for the seven domains was evaluated using a Cronbach’s alpha, with a Cronbach’s alpha of at least 0.7 considered acceptable [35].

##### Confirmatory factor analysis

We conducted confirmatory factor analysis (CFA) to assess the internal structure of the translated eHLQ. CFA was performed as the eHLQ has a prespecified factor structure. We evaluated the extent to which the items loaded on the seven hypothesized scales (i.e., the latent factors) based on the seven dimensions that the eHLQ intends to measure. CFA was performed using the R package Lavaan in R version R-3.6.1 [63]. We fitted a seven-factor CFA model allowing for correlation between latent factors. The Diagonally Weighted Least Squares estimator was used, which is the recommended estimation for ordinal data [64]. The CFA provided the standardized and unstandardized factor loadings between item responses and the underlying latent variables. In line with the original eHLQ development study, we report on the robust indexes comparative fit index (CFI), Tucker-Lewis index (TLI), Standard Root Mean Square Residual (SRMR) and Root Mean Square Measure of Approximation (RMSEA). We used the following threshold values for the test of good model fit; CFI > 0.95, TLI > 0.95, SRMR < 0.08 and RMSEA < 0.06 and the thresholds: CFI > 0.90, TLI > 0.90, and RMSEA < 0.08 as indicators for reasonable fit [65]. We deemed a item factor loading of 0.4 substantial [66]. Items that performed poorly on the above criteria were flagged. To further examine the flagged items, possible model improvements were performed.

##### Invariance testing and multi-group comparison

Based on previous studies on health literacy and eHealth literacy, and the cognitive interviews, we hypothesized that the demographic characteristics age, gender, educational background and self-reported current diagnosis may affect how the items are interpreted and thus introduce measurement invariance. Hence, we defined the following subgroups prior to performing CFA: Age ≤ 45 years versus > 45 years (median split), gender, high vs. middle and low educational background, and diagnosis yes vs. no. Of the 1650 participants, 467 (28.3%) were male, 1307 (79.2%) were highly educated, and 595 (35.9%) reported to currently have a diagnosis. To investigate measurement invariance within these subgroups we performed invariance testing. From a measurement perspective, the use of multiple-item composite scales for group comparisons depends on the demonstration that: (a) the same factor structure underlies the item responses in all groups of interest (configural invariance); (b) the factor loadings are equivalent across groups (metric invariance); and (c) item intercepts (thresholds in the case of ordered categorical variables) are also equivalent across the groups (scalar invariance) [67, 68]. We performed a series of nested model comparisons with increasingly stringent equality constraints each time. The fit of each model was compared with the fit of the previous (less restricted) model. When invariance could be obtained, we moved on to the next, more restricted model. We adhered to the rule of thumb to interpret differences in CFI of > 0.01 as significant differences [69] and maintained a significance level of *p* < 0.05. For items with insufficient endorsement (≤ 2) of the response category “completely disagree”, this category was collapsed with the “disagree” category.

We performed exploratory analyses to identify patterns of eHLQ scale scores (i.e., total observed scores for each domain) across a range of sociodemographic variables. We performed post-hoc tests for differences between groups based on gender, age, education and having a current diagnosis using independent t-tests and ANOVAs. Effect sizes were calculated using Cohen’s d with the interpretation of effect size as: small < 0.20 to 0.50; medium between 0.50 and 0.80 and large > 0.80.

#### Ethical considerations

The cognitive interview study and the FitKnip study were cleared for ethics by the Medical Ethical Review Committee of the Leiden University Medical Centre (No. 19 – 078 and P20.001, respectively). Written informed consent was obtained from each participant prior to study activities.

### Results study 2

#### Participants Demographics

Participant characteristics are depicted in Table 1. The majority was female (71%) and between 35 and 54 years old (mean (SD); 45.05 (13.28). 1556 (94.3%) of the participants had a Dutch Nationality. The large majority (78.2%) reported having finished a high education, was employed full-time (43,8%) and lived with a partner and children (35.1%) or only with a partner (33.1%). About one third (30.1%) of the participants had a BMI above 25, indicative of overweight and 18% had a BMI above 30, indicative of obesity. In total 592 (35.9%) reported having a current medical, physical or psychological diagnosis, and over one third (38.4%) had used a form of healthcare the past month. Details concerning the study design and data collection are provided elsewhere [70].

**Table 1:** Characteristics of participants in study 2 (*n* = 1650)

		***n*** ** (%)**
**Gender**		
Male		467 (28.3)
Female		1177 (71.3)
Gender neutral		6 (0.4)
**Age (years)**		
Mean ± SD		45.05 ± 13.28
18 t/m 34		447 (27.1)
35 t/m 54		776 (47.0)
55 t/m 74		406 (24.6)
75 and older		21 (1.3)
**Nationality**		
Netherlands		1556 (94.3)
Suriname		16 (1.0)
Germany		10 (0.6)
Belgium		8 (0.5)
Morocco		6 (0.4)
Other		51 (3.1)
Missing/unknown		3
**BMI (kg/m2)** ^**a**^		
Mean ± SD		25.80 ± 5.05
**Education**		
Low		58 (3.5)
Middle		285 (17.3)
High		1307 (79.2)
**Living situation**		
Living with partner		547 (33.1)
Living with partner and child(ren)		580 (35.1)
Living alone		316 (19.1)
Others		207 (12.5)
**Employment status**		
Student		58 (3.5)
Employed full-time		722 (43.8)
Employed part-time		450 (27.3)
Volunteering or retired		156 (9.5)
Unemployed. on sick leave		173 (10.5)
Other		91 (5.5)
**Self-reported diagnosis**		
Yes		592 (35.9)
No		1058 (64.1)
**Utilisation healthcare past month**		
Yes		365 (38.4)
No		1017 (61.1)

^a^1648 participants

#### Preparatory analyses

For each eHLQ item, the distribution of responses, the mean and standard deviation, and Cronbach’s alpha are reported in Additional file 2. Alpha values show acceptable to good internal consistency for all seven scales (range 0.66 to 0.80).

#### Construct validity

A seven factor CFA model was fitted to the 35 eHLQ items, allowing correlation between latent factors. Standardized factor loadings were 0.51 or higher (range 0.51 to 0.84). The CFI and TLI indicated acceptable model fit (0.936 and 0.930, respectively). The RMSEA and SRMR (0.088 and 0.085, respectively) indicated that the model fit was not acceptable. However, it is not uncommon for fit indexes to show less than optimal values with large numbers of observed variables, like in the current study. Similar values for fit indices have also been reported for the closely related Health Literacy Questionnaire [71]. Closer inspection of correlation matrices indicated substantial residual correlations between items 4, 6, 7, 20 and 26. We inspected whether goodness of fit improved when allowing correlation between these items. Goodness of fit indices improved for the CFI and SRMR, exceeding the cut-off value for good fit. Considering that item 26 (‘I use measurements about my body to help me understand my health’) showed strong residual correlation with seven other items and goodness-of fit improved, item 26 was flagged for discussion (see Table 2). Correlation matrices are provided in Additional file 2.

**Table 2:** Model fit indices for the seven-factor confirmatory factor analysis of the Dutch version of eHealth Literacy Questionnaire

	Chi square	DF	CFI	TLI	RMSEA (95% CI)	SRMR	Goodness of fit
Seven factor model	7429.201	539	0.936	0.930	0.088 (0.086 -0.09)	0.085	Acceptable fit
Seven factor model – improved model	5960.518	527	0.950	0.943	0.079 (0.077–0.081)	0.077	Good fit

*CFI* Comparative fit index, *CI* 90% confidence interval, *DF* Degrees of freedom, *RMSEA* Root mean squared error of approximation, *SRMR* Standardised root mean squared residual

#### Invariance testing

Invariance testing for subgroups based on age, gender, education and current diagnosis showed configural invariance, indicating that the overall factor structure of the eHLQ is the same between subgroups. We did not obtain full metric invariance in any of the subgroup comparisons (Table 3). Following the significant Δχ^2^ tests, we examined the univariate test scores and identified the item loadings which showed the strongest lack of invariance, indicated by larger chi-square values. Lifting between-group equality restrictions on these loadings, we obtained models of partial metric invariance: We lifted restrictions on loadings of items 15, 21, 23 and 26 for comparison of age groups; items 20, 19 and 29 for gender; items 10, 14, 22, 30 and 31 for educational level and items 11, 19, 23 and 25 for current diagnosis and. All models improved and partial metric invariance was supported, indicating that the model is partially metric invariant (see Table 3). Next, we applied between-group equality restrictions to item thresholds, which proved tenable according to the non-significant decreases in Δχ^2^ tests (Table 3). Closer examination of the noninvariant items showed comparable factor loadings between groups and standard errors below 0.1, except for item 19, hence we concluded that the items were sufficient invariant to allow comparison of scores between groups (See Additional file 3).

**Table 3:** Results of invariance testing among a priori subgroups (*n* = 1650)

	X^2^	DF	CFI	TLI	RMSEA (95% CI)	SRMR	ΔΧ^2^	ΔDF	*P*-value significance level
**Age (18–45 yr vs ≥ 45 yr)**									
Configural invariance	8040	1078	0.939	0.932	0.089	0.088			
Metric invariance	8348	1106	0.936	0.931	0.089 (0.089—0.091)	0.090	80.919	28	< .001
*Partial metric invariance* ^*a*^	*8186*	*1102*	*0.938*	*0.933*	*0.088 (0.087–0.089)*	*0.089*	*41.310*	*24*	< .05
Scalar invariance	8402	1168	0.936	0.935	0.087(0.085 – 0.088)	0.088	68.921	62	n.s
**Gender (male vs female)**									
Configural invariance	8004	1078	0.937	0.930	0.088 (0.087 -0.090)	0.089			
Metric invariance	8252	1006	0.935	0.934	0.086 (0.084—0.088)	0.089	54.740	28	< .001
*Partial metric invariance* ^*b*^	*8136*	*1103*	*0.936*	*0.93*	*0.088 (0.086—0.090)*	*0.089*	34.861	25	n.s
Scalar invariance	8237	1167	0.935	0.934	0.086 (*0.084 -0.088)*	0.089	12.720	61	n.s
**Education (low and middle vs high)**									
Configural invariance	7733	1078	0.940	0.933	0.087 (*0.085–0.088)*	0.085			
Metric invariance	8026	1106	0.937	0.933	0.087 (*0.085—0.089)*	0.087	51.676	28	< .01
*Partial metric invariance* ^*c*^	*7891*	1103	0.938	0.933	0.087 (*0.085—0.088)*	0.086	36.055	25	n.s
Scalar invariance	8068	1169	0.938	0.936	0.085 (*0.083–0.086)*	0.085	58.695	63	n.s
**Diagnosis (yes vs no)**									
Configural invariance	8080	1078	0.937	0.93	0.089 (*0.087 -0.091)*	0.088			
Metric invariance	8382	1106	0.935	0.930	0.089 (*0.088—0.091)*	0.090	73.577	28	< .001
*Partial metric invariance* ^*d*^	*8198*	1102	0.936	0.931	0.088 (*0.087—0.090)*	0.089	32.745	24	n.s
Scalar invariance	8343	1168	0.935	0.934	0.086 (*0.085—0.088)*	0.089	18.489	62	n.s

*CFI* Comparative fit index, *CI* 90% confidence interval, *DF* Degrees of freedom, *RMSEA* Root mean squared error of approximation, *SRMR* Standardised root mean squared residual, *n.s* Non-significant

^a^lifted restrictions on loadings of items 15, 21, 23 and 26

^b^lifted restrictions on loadings of items 20, 19 and 29

^c^lifted restrictions on loadings of items 10, 14, 22, 30 and 31

^d^lifted restrictions on loadings of items 11, 19, 23 and 25

##### Comparison of scores between groups

Comparison of mean scores between subgroups are presented in Table 4. The group of people with people 45 years or older consistently had lower scores than the younger age group. Statistically significant differences were found for domains ‘2. Engagement in own health’, ‘3. Ability to actively engage with digital services’ and ‘ 7. Digital services that suit individual needs, however effect sizes were small (range 0.38 to 0.12). Comparison of mean scores across educational level (low vs. high) showed moderate effect sizes on the domain scores domains ‘2. Engagement in own health’, ‘3. Ability to actively engage with digital services’, 6. ‘Access to digital services that work’ and ‘ 7. Digital services that suit individual needs’, with people with lower education scoring lower for all, except domain 4 ‘Feel safe and in control’. People with a current diagnosis scored significantly higher on domain ‘1. Using technology to process health information’, ‘2. Engagement in own health’ and ‘6. Access to digital services that work’ but again effect sizes were small (range 0.13 to 0.26).

**Table 4:** Comparison of mean domain scores between a priori subgroups (*n* = 1650)

	**1. Using technology to process health information**	**2. Engagement in own health**	**3. Ability to actively engage with digital services**	**4. Feel safe and in control**	**5. Motivated to engage with digital services**	**6. Access to digital services that work**	**7. Digital services that suit individual needs**
**Age**							
18—45 yr (*n* = 447) (ref)	2.83	3.07	3.24	2.79	2.89	2.52	2.59
≥ 45 yr (*n* = 776)	2.83	3.02*	3.06***	2.78	2.88	2.54	2.50***
Cohen’s d (95% CI)	0.00 (-0.10 -0.10)	0.12 (0.02 -0.21)	0.38 (0.28 -0.47)	0.02 (-0.08 -0.12)	0.03 (-0.07 -0.12)	-0.04 (-0.14 -0.06)	0.19 (0.09 -0.29)
**Gender**							
Men (*n* = 467) (ref)	2.82	3.02	3.18	2.78	2.96	2.52	2.55
Women (*n* = 1177)	2.83	3.06	3.14	2.79	2.86***	2.53	2.55
Cohen’s d (95% CI)	0.00 (-0.11 -0.11)	-0.10 (-0.21 – 0.00)	0.10 (-0.01 – 0.21)	-0.02 (-0.13 – 0.09)	0.26 (-0.14 – 0.08)	-0.03 (-0.14 -0.08)	0.00 (-0.10 – 0.11)
**Educational level**							
Low (*n* = 58) (ref)	2.72	2.81	2.9	2.98	2.83	2.74	2.66
Middle (*n* = 285)	2.85	2.94	3.09**	2.94	2.92	2.71	2.67
High (*n* = 1307)	2.83	3.08***	3.17***	2.74**	2.88	2.48***	2.52
Cohens’ d (low vs middle)	-0.32 (-0.60 –0.04)	-0.34 (-0.62—-0.05)	-0.44(-0.73 –0.16)	0.10 (-0.19 – 0.38)	-0.23 (-0.52 -0.05)	0.05 (-0.23 -0.33)	-0.03 (-0.31 -0.25)
Cohen’s d (low vs high)	-0.24(-0.50 – 0.02)	-0.67 (-0.93 –0.41)	-0.60(-0.86—0.34)	0.50 (0.24–0.76)	-0.14 (-0.40–0.12)	0.58(0.32–0.84)	-0.28 (0.02–0.55)
**Current diagnosis**							
Yes (*n* = 592) (ref)	2.89	3.08	3.18	2.82	2.89	2.60	2.53
No (*n* = 1058)	2.79***	3.02**	3.13	2.76*	2.89	2.48***	2.56
Cohen’s d (95% CI)	0.24 (0.14 -0.34)	0.15 (0.05–0.25)	0.10 (0.00 -0.20)	0.13 (0.02–0.23)	0.01 (-0.09 – 0.11)	0.26 (0.16 -0.36)	-0.07 (-0.17 – 0.03)

*Ref* Reference group, *CI* Confidence interval

^***^ *p* < .001, **p < .01, *p < .05

##### Data triangulation study 1 and study 2 and final revision

Evidence from both studies and all sources was collected, combined and triangulated. Table 5 presents a summary of the evidence collected for the three sources of validity evidence (i.e., test content, response process and internal structure). Overall, no large response process problems were found, the items were interpreted as intended, and the internal structure was equivalent to the original eHLQ with acceptable to good model fit indices. The items which were flagged based on the cognitive interviews were compared with the results from the CFA and invariance testing and discussed during a final consensus meeting.

**Table 5:** Summary of the three sources of validity evidence for the eHealth Literacy Questionnaire

Validity argument^a^	Evidence collected
**1. Test content**	
1.1 The items are clear and understandable to everyone without any technical jargon	No evidence of major misunderstanding observed during cognitive interviewing. The wording or tone of four items were amended based on cognitive interviews and CFA results
1.2. The number of items is appropriate and will not cause unnecessary burden on respondents	No missing values were reported in study 2, indicative of not being overly burdensome for respondents. Number of items was deemed appropriate
1.3. The eHLQ can be administered in various formats to ensure respondents with varied skills can participate	Paper-based format (study 1), face-to-face interviews (study 1) and web-based format were administered. No problems were encountered among any of the formats
1.4 The paper-based or web-based formats of items allow for easy response to items	Some issues were identified in responding to the items during cognitive interviews. The issues related to discordance in resonance with worldviews
**2. Response process**	
2.1 The response option of a four-point ordinal scale is appropriate	Four participants desired an additional response option for the items identified as problems with limited applicability
2.2. Formats of administration do not affect the cognitive process of responding to the items	Not evaluated. Prior studies show no difference in administration formats [41]
2.3 The items are understood by respondents as intended by the test developers	Twelve items showed problems in limited applicability, unclear reference and problems with wording or tone. Comparison to CFA results and discussion with the consensus team led to the revision of eight items
2.4 The items are understood in the same way by respondents as intended across subgroups	Differences in interpretation were seen during cognitive interviews based on (digital) healthcare use. Eight items were identified as having problems with ‘limited applicability’ or ‘resonance with worldview’. This observation was confirmed by invariance tests demonstrating partial invariance between groups based on current diagnosis Differences in interpretation can be the result of prior experience with digital health technology use. We recommend administers of the eHLQ to collect contextual information on prior and current eHealth use and diagnosis. Also recommend collecting eHLQ validity evidence in different settings and populations and perform invariance testing based on eHealth experience
**3. Internal structure**	
3.1 The items of each construct reflect a spectrum of the relevant construct such that the resulting score is a good indicator of the construct	Only item 26 showed strong residual correlation with seven other items, indicating that the item relates strongly to other items and the underlying latent factor
3.2 The eHLQ is a multidimensional tool consisting of seven independent constructs with 4 to 6 relevant items for each construct and such items are related only to the designated construct	CFA confirmed adequate model fit for the seven-factor model. Model and fit indices were acceptable. Standardized factor loadings were 0.51 or higher (range 0.51 to 0.84). No significant cross-loadings were identified
3.3. The eHLQ demonstrates measurement equivalence across subgroups and settings	eHLQ is partially invariant for subgroups age, gender, educational level and current diagnosis. Items displaying potential non-invariance were revised, triangulated with cognitive interview data and resulted in amendment of four items
3.4 The eHLQ produces stable and consistent results	Cronbach alpha levels were acceptable. Pre-testing was not performed

^*a*^ *validity argument adopted from Cheng *et al. (40)

Closer examination of the items contributing to non-invariance among the subgroups for current diagnosis showed that item 11 (’I often use technology to understand health problems’) had the largest contribution. Two other items were identified as ‘problem resonance worldview’ based on the cognitive interviews, indicating that people with a diagnosis probably interpret the item as referring to their own health, whereas those without a diagnosis probably interpret the item as referring to ‘health in general’. As such, interpretation of these items may depend on presence of a current diagnosis or not. Likewise, the items contributing to non-invariance between the age subgroup could be differently interpreted based on healthcare use as the older group probably had more healthcare use experience overall. Closer examination of the non-invariance among the subgroups showed that four of the five items load to domain 4 ‘feel safe and in control’. While no problems were encountered in the cognitive interviews, the observed partial invariance and inconsistency in scoring patterns could point towards a difference in interpretation.

Eight items were amended following discussion of the findings and flagged items with the consensus team and developer s. In addition, we formulated several recommendations for those using the eHLQ that can support the use, administration and interpretation of the eHLQ (see Table 6).

**Table 6:** Recommendations for future eHLQ use, score interpretation and reporting by researchers and others

1. Collect contextual information such as prior eHealth experience, and current diagnosis, depending on purpose of use and use in score interpretation.
2. Perform validity analysis in new contexts to build further validity arguments for use of the Dutch eHLQ.
3. Perform cognitive interviews when used in a new context to test if intended interpretation of data is valid for the new context and reason for testing.
4. Include description of local or national digital healthcare context, depending on context and purpose of use.

## Discussion

This paper reported on the systematic translation of the eHLQ into Dutch and initial validity evidence. We used evidence on test content, response process and internal structure to further refine and culturally adapt the Dutch eHLQ. This validity-driven approach created an in-depth understanding on content, response process and internal structure of the Dutch eHLQ. Our study builds on a well-established line of research and strengthens the continuous strand on validity evidence of the eHLQ as a global instrument to measure eHealth literacy.

The translated and culturally adapted eHLQ items were found to be highly coherent with the original intended item meanings and demonstrated good internal structure, comparable with the original eHLQ [35]. All 35 items loaded strongly or moderately on their respective factor. After one modification (i.e., allowing a residual correlations with item 26 ‘I use measurements about my body to help me understand my health’), the model showed good fit with the data. Item 26 showed similar residual correlation issues in a validity study in an Australian population [41] and in Taiwan using a Mandarin version [72]. In fact, both studies found a lower factor loading (factor loading 0.36 and 0.56 respectively) than our study (factor loading 0.61). Hence, it is unlikely that the observed validity issue with item 26 results from translation or cultural adaption, but rather is a characteristic of the item that is notable across settings and languages. We also tested invariance of item loadings and thresholds between age, gender, education and current diagnosis groups, and found that only a small subset of loadings differed between groups, indicating that the eHLQ measures largely the same construct in the same manner, in different groups.

Multi-group comparison showed that, overall, people who were younger scored higher across domains. This is in line with other literature demonstrating that older age is associated with lower eHealth literacy [73]. We also observed that people with a lower education overall scored lower than those with a higher education. This is in line with previous eHLQ studies and with the notion that, generally, people with lower education use eHealth less often [72, 73]. Interestingly, and contrasting with previous studies, in our study, people with lower education scored higher on the domain ‘feel safe and control’. At the same time, items that loaded on this domain showed metric non-invariance based on education. Hence, the higher score could potentially result from a difference in interpretation between people with low and high education, rather than reflect true differences in domain 4 ‘feel safe and control’. Future research should explore these observed differences further.

Our approach of collecting and combining the three sources of validity evidence to inform the final translation and cultural adaption of a questionnaire is a new, highly disciplined and transparent approach to validity testing, informed by contemporary validity testing theory [45]. By combining the insights from the cognitive interviews with results from CFA and invariance testing, we were able to leverage both the depth of qualitative data as well as the quantitative power of large sample analysis and psychometric evaluation methods. While cognitive interviews were successful in identifying items which demonstrated potential problems (in wording, phrasing, or resonance with world views), CFA helped to understand if and how interpretation issues may affect the internal structure. Vice versa, the qualitative data helped to interpret CFA results, such as lower standardized factor loadings in some subgroups, that may indicate interpretation difficulties. However, low factor loadings on itself do not provide information of where the problem lies. Therefore, in-depth exploration of response process and how items are interpreted using cognitive interviewing was important. Hence, with our approach, we were able to better understand the source and impact of these intricacies and make well-substantiated amendments to eight items. In addition we formulated several recommendations to support the use, administration and interpretation of the eHLQ (see Table 6).

Our approach can be considered an amalgamation of an ideographic approach (i.e., the participant is considered a unique individual with a unique life history) and a nomothetic approach (i.e., the participant is an exemplar of a population with corresponding traits). From an ideographical perspective, the qualitative component of cognitive interviewing is used to understand how items are interpreted by an individual and how this is affected by previous experiences in medical and psychosocial domains (e.g., previous positive experience in using eHealth, previous diagnosis, healthcare use, etc.). From a nomothetic perspective, items scores and latent factor structures are a result of subjects being an exemplar of a given population with (assumed) corresponding personal traits and behaviours and whose behaviour can at least partially be explained following certain rules. This strong nomothetic approach forms the foundation of psychometric evaluation, with use of standardized methods and statistical analysis as the basis. As such, our combined approach allows us to understand in more depth how items are interpreted by individuals, and to combine this information with generalized findings from the CFA to inform the final instrument. Hence, this mixed-method approach creates a conjunction between ideographic and nomothetic perspectives in instrument design and underlines the importance of considering both approaches in the understanding of complex constructs such as eHealth literacy.

An important strength of our research is that we followed a systematic, uniform translation approach and aligned our validity assessment with initial validation studies. This uniform process facilitated international comparison and helped to understand whether a validity issue has arisen during the translation process or can be considered an item characteristic and deemed acceptable. We undertook several steps to ensure validity during the translation and cultural adaption process. First, the translation process followed a rigorous translation procedure including forward and backward translations. Second, we used the Translation Integrity Protocol developed by the developers of the original instrument, using detailed specification of item intents and consensus meeting with the consensus team and developers. With this we ensured that the items in the Dutch version captured the same meaning and difficulty level compared to the original questionnaire and the subsequent translations into twenty other languages. Third, we carefully documented all steps of the process, and ensured that both the developers and people with clinical, research and linguistic expertise were engaged in the translation process. Fourth, we analysed the cognitive interviews following an analysis framework and discussed results with the consensus team.

## Limitations and strengths

A drawback of our study is that the study sample from study 2 is drawn from an existing study population. Although using an existing sample is a cost-effective sampling method and has resulted in a large sample to draw conclusions, using an existing sample has some limitations. First of all, the single administration of the eHLQ did not allow for a test–retest comparison to provide further evidence on the stability and consistency of results. Second, given that the study focuses on health budget and health apps, study participants were probably more highly sensitised to eHealth than the general population. However, given that eHLQ scores were comparable with previous eHLQ studies, it is unlikely that this has biased our results. The sample was also not fully representative in terms of educational level and nationality, with an overrepresentation of high education and Dutch nationality. Nonetheless, we encourage researchers to be aware of the multi-cultural Dutch populations when evaluating eHealth Literacy in a Dutch setting and considering the most appropriate language based on the study population. Importantly, our population included both people with a current diagnosis as well as without [72]. This increases the generalizability and applicability of our findings as the eHLQ has been developed to be used in a wide range of settings.

Another limitation may be the use of cognitive interviewing to assess response process and test content. Cognitive interviewing has been criticized and considered inappropriate for people who are less articulate and find it difficult to verbalize their thought process. Consequently, this could result in overestimation or underestimation of response difficulties (i.e., difficulties in articulation of thoughts interpreted by the investigator as response process issues or the other way around, when people are unable to accurately articulate the problems, they encounter). We tried to minimize this limitation by combining think-aloud as a primarily respondent-driven with scripted-probing as a more interviewer-driven approach. Finally, a limitation of our approach is that we have not evaluated the eight items we improved on validity. Considering the minor changes made in wording we expect that the internal structure validity will remain equivalent to the original. Nonetheless, in line with The Standards and the establishment that tests, or instruments are themselves not valid or invalid, but rather are valid for a particular use, we encourage researchers to use this initial validity evidence, build on it and always consider validity of the eHLQ in the context of the particular use and intended purpose.

## Implications for practice and research

Our findings have implications for future use of the eHLQ by policy makers, eHealth developers and researchers in understanding people’s eHealth literacy. Researchers should collect relevant contextual data (e.g., experience with technology, current diagnosis) to aid the interpretation of eHealth literacy scores and understand score differences between groups. For example, we noticed differences in the interpretation of ‘health technology’ and ‘health technology services’ depending on former experience with eHealth. Also, contextual information on current diagnosis and/or extent of healthcare usage can aid score interpretation.

Next to the use of contextual information of the individual, researchers should interpret their eHLQ scores in light of the local or national digital healthcare context (macro context) [74]. Understanding of the digital landscape from a macro perspective, in terms of the delivery, access, integration, and (inter)connectivity of systems and services is particularly important in the interpretation of scores for domain 6’ Access to digital services that work and domain 7’ Digital services that suit individual needs. While the items touch upon the maturity of healthcare systems and services, response processes may be country specific and affected by the national advancement in the health technology infrastructure (e.g., access to a national infrastructure for telehealth solutions, use of centralized health databases) [74]. Building on the existing literature that explores the link between relatable concepts such as motivation, engagement, trust, activation and health literacy, we propose that future studies should investigate the relationship between eHealth literacy and related concepts [75, 76]. In addition we suggest that future research should investigate the role of these constructs in determining health outcomes and how they can be incorporated in the design of health interventions to foster meaningful patient engagement in the digital health landscape.

The eHLQ’s large number of items makes the questionnaire less suitable for use in practice. There is a need among healthcare professionals to assess the eHealth literacy needs of their patients [77]. To address their needs, the eHealth Literacy Assessment toolkit (eHLA) was developed in parallel to the eHLQ [34]. The toolkit employs a combination of existing and newly developed scales to assess individuals’ health literacy and digital literacy across the seven dimensions of the eHLQ [78]. Our findings could inform the development of a Dutch version of the eHLA, which could assist in the implementation and evaluation of digital health technologies and services.

## Conclusion

We systematically performed and combined several procedures to generate comprehensive validity evidence of the eHLQ and conducted informed further refinement of the eHLQ. The objective of this study was to provide initial evidence on the validity and use of the eHLQ as a Dutch person-centred instrument to measure eHealth literacy, rather than to provide a complete picture of all aspects of validity. This study demonstrates that the Dutch version of the eHLQ can be considered a robust instrument which can be used by policy makers, eHealth developers and researchers to understand people’s ability to engage with and use technology so that these systems can be developed, evaluated, and redesigned to meet the eHealth Literacy need of their communities. Ultimately, this is necessary to provide appropriate support and work towards an inclusive, equitable digital healthcare landscap e.

## Supplementary Information

**Additional file 1: Multimedia Appendix 1.** Characteristics of participants in cognitive interviews (study 1).

**Additional file 2: Multimedia Appendix 2.** Descriptive statistics Dutch eHLQ items and internal consistency (*n*=1650).

**Additional file 3: Multimedia Appendix 3.** Non-invariant loadings in each of the sub-group comparisons.

**Additional file 4: Multimedia Appendix 4.** Translation, cognitive interviews and standardized factor loadings of the seven-factor models of the Dutch eHLQ.

## Data Availability

Individual de-identified participant data supporting findings of study 1 are available from corresponding author on reasonable request. The raw data that support the findings of study 2 are available from the FitKnip study but restrictions apply to the availability of these data, which were used under license for the current study, and so are not publicly available. Data are however available from the authors upon reasonable request and with permission of the FitKnip study investigators at the Leiden University Medical Centre (fitknip@lumc.nl).

## Abbreviations

CFA: Confirmatory Factor Analysis
CFI: Comparative Fit Index
DLI: Digital Literacy Instrument
eHEALS: The eHealth Literacy Scale
eHLF: eHealth Literacy Framework
eHLQ: eHealth Literacy Questionnaire
NeLL: National eHealth Living Lab
RMSEA: Root Mean Square Measure of Approximation
SRMR: Standard Root Mean Square Residual
TIP: Translation Integrity Procedure
TLI: Tucker-Lewis Index
The * Standards*: The Standards for Educational and Psychological Testing
WHO: World Health Organization
