# Supplementary material for: Translation, cultural adaptation and validity assessment of the Dutch version of the eHealth Literacy Questionnaire: a mixed-method approach
Source: BMC Public Health. 2023 May 30;23:1006. doi: 10.1186/s12889-023-15869-4 (PMC10227819; doi:10.1186/s12889-023-15869-4)
Supplement: Supplementary file 1 — Additional file 1: Multimedia Appendix 1. Characteristics of participants in cognitive interviews (study 1). [file 12889_2023_15869_MOESM1_ESM.docx]

**Multimedia Appendix 1. Characteristics of participants in cognitive interviews (study 1)**

|  | Gender | Age | Education | professionally involved in healthcare | technologie affiniteit | previous experience with eHealth |
| --- | --- | --- | --- | --- | --- | --- |
| CI-001 | M | 67 | MBO | no | nee | bloodpressure device, digitale thermometer |
| CI-002 | M | 78 | WO | yes | yes | Computer, smartphone, online patientportaal |
| CI-003 | V | 36 | WO | no | no | Via son; computer, notebook, tablet, smartphone |
| CI-004 | M | 73 | HBO? | yes | no | internet, laptop, normale telefoon |
| CI-005 | M | 62 | MBO | no | yes | computer notebook, tablet, smartphone, apps, wearable |
| CI-006 | V | 38 | MBO | no | yes | apps, websites, online patientenportal |
| CI-007 | V | 27 | WO | no | no | internet, thuisarts.nl, online patientenportal GP. |
| CI-008 | V |  | WO | yes | no | internet, bloodpressure device, thuisarts.nl |
| CI-009 | M | 39 | MBO | no | no | pedometer, hartslagmeter, app voor physical activity, internet |
| CI-010 | M | 55 | WO | no | yes | laptop, internet, smartphone |
| CI-011 | M | 61 | Middelbare school | no | yes | pedometer, vragenlijst opzoeken, google |
| CI-012 | M | 71 | WO | no |  | pollenmeter,online questionnaires |
| CI-013 | M | 1951 | MBO | no | yes | MedApp, smartwatch, slimme weegschaal, hartkastjes (wil informatie van monitor); veel ervaring, bewust mee bezig |
| CI-014 | M | 1951 | HBO | no | yes | bloodpressure device, smart devices, deelname panneldiscussie eHealth artsen CV patiënten. |

M: male, F:Female
