# Supplementary material for: Translation, cultural adaptation and validity assessment of the Dutch version of the eHealth Literacy Questionnaire: a mixed-method approach
Source: BMC Public Health. 2023 May 30;23:1006. doi: 10.1186/s12889-023-15869-4 (PMC10227819; doi:10.1186/s12889-023-15869-4)
Supplement: Supplementary file 2 — Additional file 2: Multimedia Appendix 2. Descriptive statistics Dutch eHLQ items and internal consistency (n=1650). [file 12889_2023_15869_MOESM2_ESM.docx]

| 1. Using technology to process health information – Cronbach’s Alpha = 0.67   **Multimedia Appendix 2 :** Descriptive statistics Dutch eHLQ items and internal consistency (n=1650) | | | | | | |  |  |
| --- | --- | --- | --- | --- | --- | --- | --- | --- |
| Item nr | **Domain** | **Item** | **Mean** | **SD** | **Stongly disagree (1)** | **Disagree (2)** | **Agree (3)** | **Strongly agree (4)** |
| x7 | 1 | I use technology to find … | 3,26 | 0,550 | 0.3 | 4.6 | 63.9 | 31.2 |
| x11 | 1 | I often use technology … | 3,00 | 0,617 | 0.8 | 16.8 | 64.4 | 18.1 |
| x13 | 1 | Technology helps me … | 2,87 | 0,599 | 1.5 | 21.1 | 66.7 | 10.7 |
| x20 | 1 | I use technology to share … | 2,45 | 0,742 | 9.2 | 42.4 | 42.5 | 5.9 |
| x25 | 1 | I use technology to organise … | 2,56 | 0,698 | 5.5 | 40.1 | 47.9 | 6.5 |
| 1. Engagement in own health – Alpha = 0.68 | | | | | | |  |  |
| x5 | 2 | The knowledge I have helps me… | 3,12 | 0,592 | 0.2 | 11.3 | 64.2 | 24.2 |
| x12 | 2 | I have enough information … | 3,10 | 0,568 | 0.3 | 10.7 | 67.7 | 21.3 |
| x15 | 2 | I understand medical results … | 2,96 | 0,633 | 1.1 | 18.8 | 63.0 | 17.0 |
| x21 | 2 | Overall, I understand how … | 3,24 | 0,544 | 0.4 | 4.6 | 65.8 | 29.3 |
| x26 | 2 | I use measurements about my body … | 2,80 | 0,702 | 4.1 | 23.9 | 59.3 | 12.6 |
| 1. Ability to actively engage with digital services – Cronbach’s Alpha = 0.79 | | | | | | |  |  |
| x4 | 3 | I know how to use technology to get … | 3,12 | 0,624 | 1.5 | 9.8 | 64.1 | 24.7 |
| x6 | 3 | I know how to make technology … | 3,08 | 0,593 | 0.4 | 13.0 | 65.3 | 21.3 |
| x8 | 3 | I can enter data into health … | 3,02 | 0,716 | 2.7 | 16.7 | 56.7 | 23.9 |
| x17 | 3 | I quickly learn how to find my way … | 3,30 | 0,611 | 0.2 | 7.5 | 54.7 | 37.5 |
| x32 | 3 | I easily learn to use new … | 3,24 | 0,590 | 0.4 | 6.9 | 60.6 | 32.1 |
| 1. Feel safe and in control – Cronbach’s Alpha = 0.80 | | | | | | |  |  |
| x1 | 4 | I am sure that my health data … | 2,86 | 0,712 | 2.8 | 25.1 | 55.7 | 16.4 |
| x10 | 4 | My electronic healthcare data … | 2,78 | 0,609 | 2.4 | 24.8 | 65.3 | 7.6 |
| x14 | 4 | I have a clear understanding … | 2,65 | 0,681 | 3.4 | 36.4 | 51.8 | 8.4 |
| x22 | 4 | I am sure that only authorised people … | 2,58 | 0,703 | 4.5 | 40.9 | 46.6 | 7.9 |
| x30 | 4 | I am confident that healthcare providers … | 3,05 | 0,590 | 1.4 | 11.0 | 69.2 | 18.5 |
| 1. Motivated to engage with digital services – Cronbach’s Alpha = 0.67 | | | | | | |  |  |
| x2 | 5 | Technology makes me feel actively … | 2,89 | 0,599 | 1.1 | 20.8 | 66.2 | 11.9 |
| x19 | 5 | I find technology helps me … | 2,92 | 0,579 | 1.3 | 17.5 | 69.6 | 11.6 |
| x24 | 5 | I find I get better services … | 2,69 | 0,666 | 3.0 | 33.8 | 54.8 | 8.5 |
| x27 | 5 | Technology improves my communication … | 2,74 | 0,664 | 3.3 | 28.5 | 59.0 | 9.2 |
| x35 | 5 | I find technology useful for monitoring … | 3,21 | 0,545 | 0.3 | 5.8 | 66.9 | 27.0 |
| 1. Access to digital services that work – Cronbach’s Alpha = 0.71 | | | | | | |  |  |
| x3 | 6 | Information about my health … | 2,68 | 0,774 | 5.6 | 34.4 | 46.5 | 13.4 |
| x9 | 6 | My healthcare providers deliver … | 2,70 | 0,746 | 6.5 | 27.9 | 54.8 | 10.8 |
| x16 | 6 | My health data are available … | 2,35 | 0,767 | 11.2 | 49.7 | 32.2 | 6.8 |
| x23 | 6 | All the health technology … | 2,04 | 0,604 | 15.6 | 66.0 | 17.6 | 0.8 |
| x29 | 6 | Most of my healthcare providers can … | 2,68 | 0,707 | 5.2 | 30.3 | 55.6 | 8.9 |
| x34 | 6 | I have access to health technology … | 2,71 | 0,648 | 3.6 | 28.8 | 60.4 | 7.2 |
| 1. Digital services that suit individual needs – Cronbach’s Alpha = 0.74 | | | | | | |  |  |
| x18 | 7 | I find that health technology services adapt … | 2,51 | 0,663 | 4.7 | 44.5 | 46.0 | 4.8 |
| x28 | 7 | I find health technology services seem to … | 2,39 | 0,632 | 6.2 | 50.7 | 41.2 | 1.9 |
| x31 | 7 | I find health technology services are provided … | 2,54 | 0,631 | 4.0 | 41.2 | 51.3 | 3.5 |
| x33 | 7 | Health technology services provide me … | 2,76 | 0,635 | 2.1 | 28.9 | 60.1 | 8.8 |

Items are truncated. Please contact the authors for full items.
