# Supplementary material for: Translation, cultural adaptation and validity assessment of the Dutch version of the eHealth Literacy Questionnaire: a mixed-method approach
Source: BMC Public Health. 2023 May 30;23:1006. doi: 10.1186/s12889-023-15869-4 (PMC10227819; doi:10.1186/s12889-023-15869-4)
Supplement: Supplementary file 3 — Additional file 3: Multimedia Appendix 3. Non-invariant loadings in each of the sub-group comparisons. [file 12889_2023_15869_MOESM3_ESM.docx]

**Multimedia Appendix 3 . Non-invariant loadings in each of the sub-group comparisons.**

| **Item** | **Estimated Loading** | **Standard error** | **Standardized loading** |
| --- | --- | --- | --- |
| Age (18-45 yr vs ≥ 45 yr ) |  |  |  |
| Item 15 | 0.753 | 0.042 | 0.602 |
|  | 0.884 | 0.044 | 0.667 |
| Item 21 | 0.821 | 0.040 | 0.657 |
|  | 0.651 | 0.048 | 0.491 |
| Item 23 | *0.867* | *0.092* | 0.423 |
|  | *1.053* | *0.059* | 0.605 |
| Item 26 | 0.663 | 0.050 | 0.531 |
|  | 0.907 | 0.054 | 0.684 |
| Gender (male vs female) |  |  |  |
| Item 20 | 0.890 | 0.048 | 0.609 |
|  | 0.696 | 0.053 | 0.550 |
| Item 19 | *1.273* | *0.084* | *0.654* |
|  | 1.546 | 0.151 | 0.753 |
| Item 29 | 0.915 | 0.066 | 0.472 |
|  | 1.075 | 0.089 | 0.598 |
| Education (low and middle vs high) |  |  |  |
| Item 10 | 1.199 | 0.078 | 0.769 |
|  | 1.328 | 0.046 | 0.857 |
| Item 14 | 1.284 | 0.096 | 0.824 |
|  | 1.079 | 0.051 | 0.696 |
| Item 22 | 1.237 | 0.082 | 0.793 |
|  | 1.370 | 0.049 | 0.883 |
| Item 30 | 1.276 | 0.092 | 0.818 |
|  | 1.050 | 0.050 | 0.677 |
| Item 31 | 0.917 | 0.069 | 0.667 |
|  | 1.295 | 0.054 | 0.771 |
| Current diagnosis (yes vs no) |  |  |  |
| Item 11 | 0.878 | 0.045 | 0.667 |
|  | 0.674 | 0.050 | 0.474 |
| Item 19 | 1.150 | 0.097 | 0.621 |
|  | 1.492 | 0.104 | 0.732 |
| Item 23 | 0.934 | 0.093 | 0.451 |
|  | 1.033 | 0.066 | 0.564 |
| Item 25 | 0.713 | 0.049 | 0.542 |
|  | 0.659 | 0.048 | 0.655 |
