# Supplementary material for: Translation, cultural adaptation and validity assessment of the Dutch version of the eHealth Literacy Questionnaire: a mixed-method approach
Source: BMC Public Health. 2023 May 30;23:1006. doi: 10.1186/s12889-023-15869-4 (PMC10227819; doi:10.1186/s12889-023-15869-4)
Supplement: Supplementary file 4 — Additional file 4: Multimedia Appendix 4. Translation, cognitive interviews and standardized factor loadings of the seven-factor models of the Dutch eHLQ. [file 12889_2023_15869_MOESM4_ESM.docx]

| **Item Nr.**  **Multimedia Appendix 4. Translation, cognitive interviews and standardized factor loadings of the seven-factor models of the Dutch version of eHealth Literacy Questionnaire (eHLQ)** | **Original** | **Pre-test translation** | **Problem type cognitive interview** | **CFA Standardized Factor loading** | **Amendments in translation** |
| --- | --- | --- | --- | --- | --- |
| **Domain 1. using technology to process health information** | | | | | |
| 7 | I use technology to find … | Ik gebruik technologie om … | no problem | 0.713 |  |
| 11 | I often use technology … | Ik gebruik vaak technologie om **mijn** gezondheidsproblemen te begrijpen | unclear reference | 0.551 | Ik gebruik vaak technologie om problemen met **mijn** gezondheid te begrijpen |
| 13 | Technology helps me … | Technologie helpt mij .. | resonance worldview | 0.637 |  |
| 20 | I use technology to share … | Ik gebruik technologie om … | unclear reference | 0.591 | Ik gebruik technologie om informatie over mijn gezondheid **met anderen** te delen |
| 25 | I use technology to organise … | Ik gebruik technologie om informatie over mijn gezondheid te ordenen | wording/tone | 0.615 * | Ik gebruik technologie om informatie over mijn gezondheid **bij te houden** |
| **Domain 2. understanding of health concepts and language** | | | | | |
| 5 | The knowledge I have helps me… | Door mijn kennis kan ik goed meepraten over gezondheid | no problem | 0.775 |  |
| 12 | I have enough information … | Ik heb genoeg informatie om mee te kunnen praten over mijn gezondheid | no problem? | 0.796 |  |
| 15 | I understand medical results … | Ik begrijp de uitslagen van mijn medische onderzoeken | item found out of place /unclear reference | 0.632 |  |
| 21 | Overall, I understand how … | Ik begrijp in grote lijnen hoe mijn lichaam werkt | No problem | 0.575 |  |
| 26 | I use measurements about my body … | Ik gebruik metingen met betrekking tot mijn lichaam om mijn gezondheid te begrijpen | No problem | 0.612 |  |
| **Domain 3. ability to actively engage with digital services** | | | | | |
| 4 | I know how to use technology to get … | Ik weet hoe ik technologie moet gebruiken om aan de informatie over gezondheid te komen die ik nodig heb | no problem | 0.721 |  |
| 6 | I know how to make technology … | Ik weet hoe ik technologie nuttig toe kan passen | Wording/tone | 0.787 | Ik weet hoe ik technologie **in mijn voordeel kan gebruiken** |
| 8 | I can enter data into health … | Ik kan gegevens invoeren in zorgtechnologieën | unclear respone | 0.689 | Ik kan gegevens invoeren in **digitale gezondheidsdiensten** |
| 17 | I quickly learn how to find my way … | Ik leer snel omgaan met nieuwe technologie | No problem | 0.850 |  |
| 32 | I easily learn to use new … | Ik leer gemakkelijk nieuwe zorgtechnologie te gebruiken | No problem | 0.876 |  |
| **Domain 4. feel safe and in control** | | | | | |
| 1 | I am sure that my health data … | Ik weet zeker dat de gegevens over mijn gezondheid alleen worden gebruikt door de mensen voor wie ze bedoeld zijn | No problem | 0.660 |  |
| 10 | My electronic healthcare data … | De elektronische gegevens over mijn gezondheid zijn veilig opgeslagen | No problem | 0.841 |  |
| 14 | I have a clear understanding … | Ik begrijp goed hoe zorgverleners mijn gegevens gebruiken | no problem | 0.715 |  |
| 22 | I am sure that only authorised people … | Ik weet zeker dat alleen bevoegde personen toegang hebben tot gegevens over mijn gezondheid | No problem | 0.878 |  |
| 30 | I am confident that healthcare providers … | Ik vertrouw erop dat zorgverleners goed omgaan met mijn gegevens | No problem | 0.704 |  |
| **Domain 5. motivated to engage with digital services** | | | | | |
| 2 | Technology makes me feel actively … | Door technologie voel ik me actief betrokken bij mijn gezondheid | No problem | 0.509 |  |
| 19 | I find technology helps me … | Ik vind dat technologie mij helpt om op mijn gezondheid te passen | wording/tone | 0.687 | Ik vind dat technologie mij helpt om **voor** mijn gezondheid **te zorgen** |
| 24 | I find I get better services … | Ik vind dat mijn zorgverleners mij beter van dienst zijn als ik technologie gebruik | No problem | 0.589 |  |
| 27 | Technology improves my communication … | Technologie verbetert mijn communicatie met zorgverleners | No problem | 0.671 |  |
| 35 | I find technology useful for monitoring … | Ik vind technologie nuttig bij het in de gaten houden van mijn gezondheid | wording/tone | 0.659 | Ik vind technologie nuttig bij **het monitoren** van mijn gezondheid |
| **Domain 6. access to digital services that work** | | | | | |
| 3 | Information about my health … | Informatie over mijn gezondheid is altijd beschikbaar voor de mensen die het nodig hebben | problem resonance wordview/ unclear response | 0.527 |  |
| 9 | My healthcare providers deliver … | Mijn zorgverleners bieden diensten aan die voor mij toegankelijk zijn via technologie | limited applicability | 0.613 |  |
| 16 | My health data are available … | Ik kan bij de gegevens over mijn gezondheid, waar ik ook ben | problem resonance worldview | 0.686 |  |
| 23 | All the health technology … | Alle zorgtechnologie die ik gebruik werkt samen | problem resonance worldview, wording | 0.520 | Alle zorgtechnologie die ik gebruik werkt **onderling** samen |
| 29 | Most of my healthcare providers can … | De meeste van mijn zorgverleners kan ik bereiken via technologie | No problem | 0.509 |  |
| 34 | I have access to health technology … | Ik heb toegang tot digitale gezondheidsdiensten die werken | problem resonance worldview | 0.750 |  |
| **Domain 7.** **digital services that suit individual needs** | | | | | |
| 18 | I find that health technology services adapt … | Ik vind dat digitale gezondheidsdiensten zich aanpassen aan mijn vaardigheden | problem resonance worldview | 0.623 |  |
| 28 | I find health technology services seem to … | Ik vind dat digitale gezondheidsdiensten zich lijken aan te passen aan mijn persoonlijke behoeftes | problem resonance worldview | 0.674 |  |
| 31 | I find health technology services are provided … | Ik vind dat digitale gezondheidsdiensten mij worden aangeboden op een manier die bij me past | problem resonance worldview | 0.756 |  |
| 33 | Health technology services provide me … | Door digitale gezondheidsdiensten kom ik gemakkelijk aan wat ik nodig heb | No problem | 0.809 |  |

* Strong residual correlations with items 4, 6, 7 and 20 observed

CFA: Confirmatory Factor analysis
